# Genome assembly of *Danaus chrysippus* and comparison with the Monarch *Danaus plexippus*

**DOI:** 10.1093/g3journal/jkab449

**Published:** 2021-12-31

**Authors:** Kumar Saurabh Singh, Rishi De-Kayne, Kennedy Saitoti Omufwoko, Dino J Martins, Chris Bass, Richard ffrench-Constant, Simon H Martin

**Affiliations:** 1 Bioinformatics Group, Wageningen University, Wageningen 6708 PB, The Netherlands; 2 Institute of Evolutionary Biology, University of Edinburgh, Edinburgh EH9 3FL, UK; 3 Department of Ecology and Evolutionary Biology, Princeton University, Princeton, NJ 08544, USA; 4 Mpala Research Centre, Nanyuki, P O Box 555 10400, Kenya; 5 Centre for Ecology and Conservation, University of Exeter, Penryn TR10 9FE, UK

**Keywords:** African Monarch, African Queen, Plain Tiger, repeat content, intron length, genome size, butterfly

## Abstract

Milkweed butterflies in the genus *Danaus* are studied in a diverse range of research fields including the neurobiology of migration, biochemistry of plant detoxification, host–parasite interactions, evolution of sex chromosomes, and speciation. We have assembled a nearly chromosomal genome for *Danaus chrysippus* (known as the African Monarch, African Queen, and Plain Tiger) using long-read sequencing data. This species is of particular interest for the study of genome structural change and its consequences for evolution. Comparison with the genome of the North American Monarch *Danaus plexippus* reveals generally strong synteny but highlights 3 inversion differences. The 3 chromosomes involved were previously found to carry peaks of intraspecific differentiation in *D. chrysippus* in Africa, suggesting that these inversions may be polymorphic and associated with local adaptation. The *D. chrysippus* genome is over 40% larger than that of *D. plexippus*, and nearly all of the additional ∼100 Megabases of DNA comprises repeats. Future comparative genomic studies within this genus will shed light on the evolution of genome architecture.

## Introduction

The genus *Danaus* is perhaps best known for the iconic Monarch butterfly *Danaus plexippus* and its extraordinary migrations in North America. Genomic studies of the Monarch have shed light on host plant detoxification (Tan et al. 2019), sex chromosome evolution (Mongue et al. 2017; Gu et al. 2019), and the genetic basis of migratory behavior (Zhan et al. 2014). Its relative *Danaus chrysippus* is found throughout Africa, the Mediterranean, and south Asia, and is known as the African Monarch, African Queen, and Plain Tiger butterfly in different parts of its range. *Danauschrysippus* is emerging as a useful study system in evolutionary genomics. Several subspecies of *D. chrysippus* with distinct warning patterns occupy distinct geographic ranges separated by broad hybrid zones (Smith et al. 1997; Lushai et al. 2003). Patterns of genetic differentiation suggest a role for chromosomal rearrangements in maintaining these differences (Martin et al. 2020). In the east African hybrid zone, a neo-W sex chromosome has emerged in the past few thousand years and is associated with infection by a male-killing endosymbiont *Spiroplasma* (Smith et al. 2016; Martin et al. 2020). This species therefore has great potential for future research on the evolutionary impacts of genome structural change.

Here, we describe the generation of a chromosome-level assembly for *D. chrysippus* based on Pacific Biosciences long-read sequencing data. This serves to replace a previous reference genome based on short-read sequences and mate-pair libraries, which had low contiguity (N50 = 0.63 Mb, Martin et al. 2020). Our new assembly has an N50 of 11.45 Mb. Nineteen of the 30 chromosomes are represented by a single contig and the remaining 11 by 2 contigs each. At 354 Mb, this genome is average in size for a butterfly, but about 1.4 times larger than that of *D. plexippus* (∼250 Mb). Comparative analyses indicate that this difference is largely explained by increased repeat content, but *D. chrysippus* also has larger introns, implying that these species have experienced different selection pressures acting on nonessential DNA.

## Materials and methods

### DNA sequencing

High-molecular-weight DNA was extracted from a single female pupa from a captive butterfly stock using the Qiagen Blood & Cell Culture DNA Mini Kit following the manufacturer’s guidelines. Long-read Pacific Biosciences sequencing was performed using 7 PacBio Sequel SMRT cells on the Sequel platform, yielding approximately 9.7 gigabases (Gb) per SMRT cell. The 3.8 million PacBio reads totaled 67.6 Gb and had an N50 of 27.3 kb. In addition, we generated Illumina sequencing data for the same individual on the Novaseq 600 platform (118 million paired-end reads of 150 bp with an insert size of 350 bp) totaling 35 Gb.

### Genome assembly

We assembled the long reads using both Canu (Koren et al. 2017) and Falcon (Chin et al. 2016) and then merged these assemblies to maximize the genome completeness using quickmerge -v 0.3 (Chakraborty et al. 2016). Redundant contigs or haplotigs were removed using Purge_haplotigs -v 1.0.4 (Roach et al. 2018) with the *-align_cov* (Percent cutoff for identifying a contig as haplotig) value of 65. Before merging, assemblies were polished iteratively using 3 rounds of Pilon -v 1.22 in diploid mode (Walker et al. 2014; using a trimmed version of the short-read data; reads were trimmed using Trim_Galore -v 0.4.0; Krueger 2012), and Racon -v 1.3.1 (Vaser et al. 2017; using the long-read data). Illumina and PacBio raw reads are archived under European Nucleotide Archive project accession: PRJEB47812.

### Whole-genome alignment and synteny assessment

To assess synteny and putatively assign contigs to chromosomes, we aligned the *D. chrysippus* assembly to 2 *D.plexippus* assemblies: “Dplex_v4,” a chromosome-level assembly produced by scaffolding 4,115 scaffolds using chromatin conformation (Hi-C) data (GCA_009731565.1; Gu et al. 2019) and “MEX_DaPlex,” a long-read based assembly consisting of 66 scaffolds, of which 38 (97% of total sequence) have been assigned to chromosomes (GCA_018135715.1; Ranz et al. 2021). Alignments were generated using both MUMmer’s nucmer tool version 3.1 (Marçais et al. 2018), with default parameters except the “maxGap” parameter set to 1,000, and with minimap2 v2.17 (Li 2018), using the “asm20” parameter preset, designed for whole-genome alignment of species with sequence divergence below 20%. Nucmer alignments were explored using the interactive alignment visualization tool Dot (https://github.com/dnanexus/dot) and final alignment plots based on minimap2 alignments were generated using Asynt (https://github.com/simonhmartin/asynt version 0.1).

### Correcting putative misassemblies

Visualization of whole-genome alignment to both *D.plexippus* assemblies (described above) revealed 2 putatively misassembled contigs that had portions aligning confidently to 2 different chromosomes. Although these could theoretically represent real translocation or fusion products, we took the conservative decision to split these contigs, as additional long-read assemblies generated in a related study (Kim et al. 2021) showed no evidence for translocations or fusions. Optimal split points were identified by visual inspection of the alignments, as well as additional BLASTn alignments made between the 2 genomes. The original unsplit assembly, along with details of split points, is available at https://doi.org/10.5281/zenodo.5731560.

Despite having performed automated removal of redundant contigs using Purge_haplotigs, visual exploration of the alignments identified a further 4 contigs that appeared to be redundant (i.e. representing a part of the genome already represented by a larger contig). These included one of the split products described above. To confirm this, we aligned the Illumina reads for the assembled individual back to the assembly using BWA MEM (Li and Durbin 2010) using default parameters, and computed read depth using Samtools depth (Li et al. 2009). Visualization of median read depth averaged in 50-kb windows confirmed that these 4 contigs had 50% depth, so they were removed from the assembly. Finally, 2 contigs included portions that appeared to be redundant in the alignments as well as read depth plots. These were therefore split at the point in the alignment where the redundancy began, and the redundant fragment was removed from the final assembly. The full original assembly, along with details of all splits and portions retained to produce the final Dchry2.2 assembly, is available at https://doi.org/10.5281/zenodo.5731560. To assess the base-level accuracy of our assembly, we calculated the consensus quality (QV), comparing the frequency of k-mers present in the raw Illumina reads with those present across the final assembly (all 83 contigs) using Merqury v.1.3 (Rhie et al. 2020).

### Repeat annotation

To assess the repeat content of the assembly, the genome was masked using a custom repeat library. First, a repeat library was produced using the finished genome assembly, using RepeatModeler v2.0.1 (Smit and Hubley 2008), and this library was then combined with a broad Lepidoptera repeat database extracted using RepeatMasker v.4.1.0 (Smit et al. 2015). Repeat masking of the genome was then carried out using RepeatMasker (Smit et al. 2015). To determine the prevalence of expanding transposable element families within *D.chrysippus*, the scripts calDivergenceFromAlign.pl and createRepeatLandscape.pl from RepeatModeler (Smit and Hubley 2008) were used to produce a repeat landscape for the assembly. To facilitate a comparison with other *Danaus* genome assemblies, this repeat masking process was repeated using the same custom repeat library for 2 well-assembled *D.plexippus* genome assemblies [NCBI accessions GCF_009731565.1 and GCA_018135715.1 (Ranz et al. 2021)]. The resulting soft masked assemblies were then used for genome annotation.

### Gene annotation

Due to a lack of RNAseq data, a preliminary genome annotation was carried out using 2 protein sets from the close relative to *D. chrysippus*, *D. plexippus*, the Monarch butterfly. This combined protein set was produced by collating protein information from 2 different, *D. plexippus* assemblies, the first a proteome downloaded from uniprot under the accession UP000596680 (associated with the Dplex_v4 assembly), and the second taken by extracting amino acid sequences from the annotation of the “MEX_DaPlex” *D.plexippus* assembly GCF_009731565.1 (Ranz et al. 2021). Both protein sets had high Benchmarking Universal Single-Copy Orthologs (BUSCO) scores, indicative of high-quality annotation (Simão et al. 2015). This combined protein set was then used as input for the BRAKER2 pipeline (Brůna et al. 2021) to annotate each of the 3 soft masked genome assemblies produced above (specifying –gff3 –softmasking –prot_seq=protein_set.fasta –prg=gth –gth2traingenes –trainFromGth). GenomeTools (Gremme et al. 2013) was then used to sort and tidy the annotation output (gt gff –sort –tidy –retainids –fixregionboundaries) and calculate summary statistics of the annotation (gt stat –genelengthdistri –genescoredistri –exonlengthdistri –exonnumberdistri –intronlengthdistri –cdslengthdistri). Functional annotation for the resulting *D.chrysippus* protein set was carried out using Pannzer2 (Törönen et al. 2018). To determine variation in intron and exon length between *D. chrysippus* and *D. plexippus*, introns and exons were extracted from our corresponding annotation file for each of the 3 assemblies.

### Genome comparison and assembly validation

To assess the quality of annotation, BUSCO scores were calculated for both the full *D. chrysippus* assembly and the protein sequences resulting from annotation specifying the insecta_odb10 lineage BUSCO set in BUSCO v.5.0.0 (Simão et al. 2015). Additionally, the annotation was compared against those of both published *D. plexippus* annotations. To compare each of the assemblies, and in turn, the consistency of genome structure across *Danaus* species we plotted the distribution of intron lengths for annotations from each of the 3 assemblies. This was carried out by extracting introns and exons from the longest annotated transcript for each gene within each of the annotations (using the BRAKER2 re-annotations for both *D. plexippus* assemblies to ensure lengths were comparable across assemblies).

## Results and Discussion

### Genome assembly

**Fig. 1.**
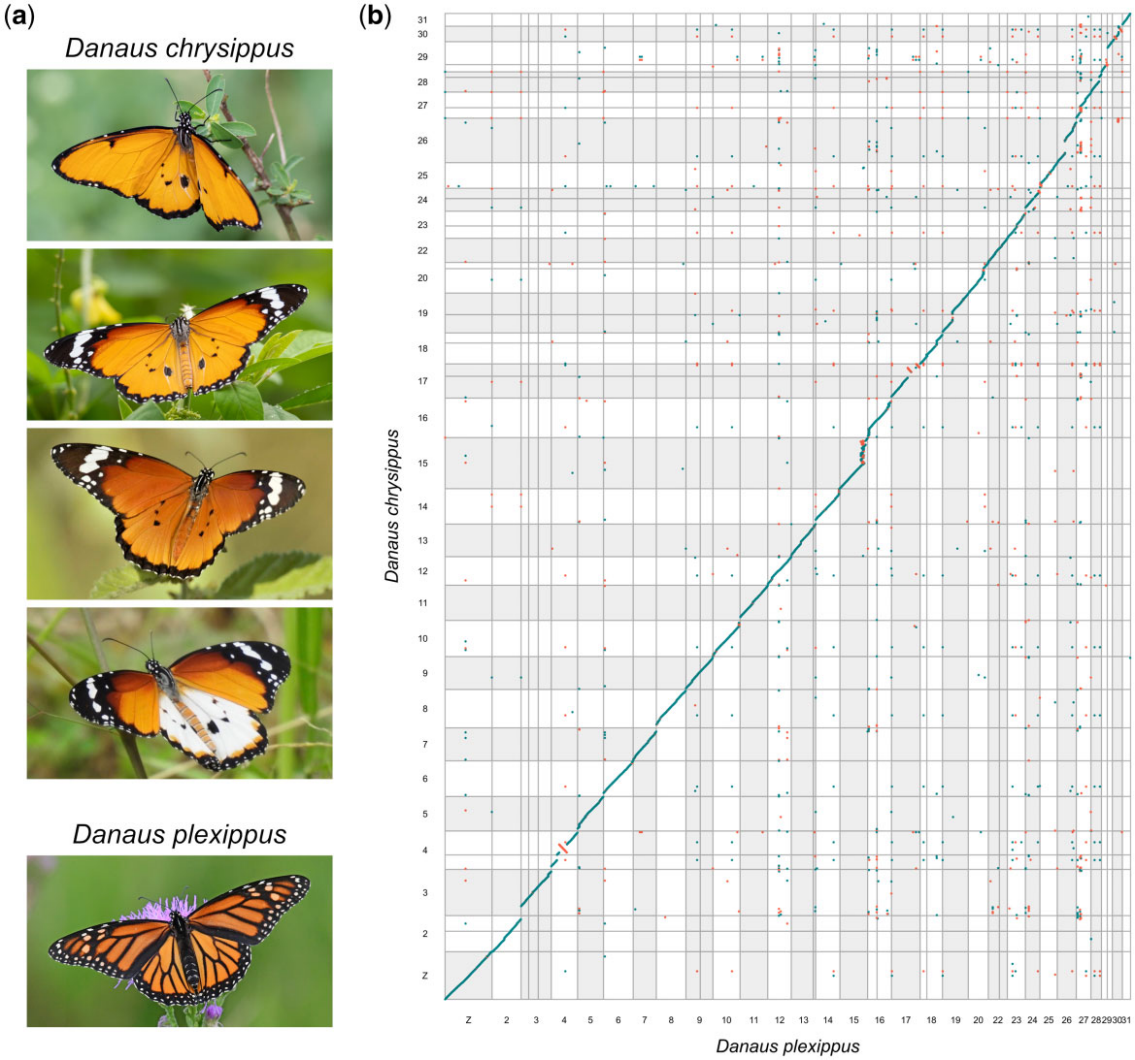
a) Four color morphs of *D. chrysippus* (above) and *D. plexippus* (below). b) Whole-genome alignment between *D. chrysippus* and *D. plexippus* (MEX_DaPlex assembly). Points represent minimap2 alignments greater than 5 kb in length. Alignments in the same orientation are shown in turquoise and those in the reverse orientation are shown in red. Only contigs that were confidently assigned to chromosomes (97% of the total in both assemblies) are included. Alternating grey and white bars indicate separate chromosomes. See Supplementary Fig. 1 for the same plot including contig/scaffold labels. Butterfly images from top to bottom by Forest Jarvis (CC-BY-NC), Paul Dickson (CC-BY-NC), Claude Martin, Steven Schulting (CC-BY-NC), and Edward Perry IV (CC-BY-NC).

In total, 67.6 Gb of long-read data was assembled into 83 contigs. Manual splitting of 3 putatively misassembled contigs and removal of several remaining redundant fragments (see *Materials and Methods*) left 83 contigs with an N50 of 11.45 Mb and L50 of 13 contigs, giving a total genome size of 354 Mb. Alignment with 2 different *D. plexippus* assemblies allowed us to confidently assign 41 contigs representing 97% of the sequence length to chromosomes (Fig. 1). Of the 30 *D. chrysippus* chromosomes, 19 are represented by a single contig and the rest by 2 contigs each. The contiguity of our assembly is therefore comparable to that of the *D. plexippus* “MEX_DaPlex” assembly (Ranz et al. 2021), for which 38 out of 66 scaffolds (97% of the assembly) were assigned to chromosomes, of which 23 are represented by a single scaffold. Among the 42 *D. chrysippus* contigs that were not assigned to a chromosome (3% of the genome), it is possible that some represent fragments of the female-specific W chromosome. However, given that butterfly W chromosomes are highly repetitive and have low interspecific homology (Lewis et al. 2021), further work comparing male and female genomes is required to test this hypothesis. The genome-wide consensus quality of the assembly (QV; representing a log-scaled probability of error for each base in our assembly) was 36.2373, suggesting a high level of accuracy (equating to an accuracy between 99.9% and 99.99%).

### Synteny and genome size comparison

The genomes of *D. chrysippus* and *D. plexippus* are largely syntenic (Fig. 1). Our assembly supports the earlier finding that the Z sex chromosome of *Danaus* species represents a fusion between the ancestral lepidopteran Z chromosome and autosome 21, which occurred in a recent ancestor of the genus (Mongue et al. 2017). We numbered chromosomes according to their homologs in the most recent *D. Plexippus* assembly (Ranz et al. 2021), which follows the growing convention of using the chromosome numbering system introduced for *Melitaea cinxia*, the first assembled lepidopteran genome that retains the ancestral karyotype of 31 (Ahola et al. 2014; Cicconardi et al. 2021; Lewis et al. 2021; Ranz et al. 2021). As such, the *Danaus* karyotype lacks an autosome 21, as this is now part of the Z sex chromosome.

Several putative inversion differences can be identified between the 2 *Danaus* species, most notably on chromosomes 4, 17, and 30 (Fig. 1). We note that all 3 of these chromosomes were found to carry sharp peaks of intraspecific differentiation between subspecies of *D. chrysippus* in Africa, against a background of very low genetic differentiation (Martin et al. 2020), suggesting that these putative inversions may be polymorphic and associated with local adaptation in *D. chrysippus*. In addition, chromosomes 15, 26, and 29 all carry large duplicated/repetitive portions relative to *D. plexippus*. One of these, on chromosome 15, was identified previously as the site of a large expansion in gene copy number through multiple duplications and is associated with subspecies differentiation and color pattern variation in *D. chrysippus* (Martin et al. 2020). Further work to dissect this genomic region and compare chromosome structure among *D. chrysippus* subspecies is ongoing.

**Fig. 2.**
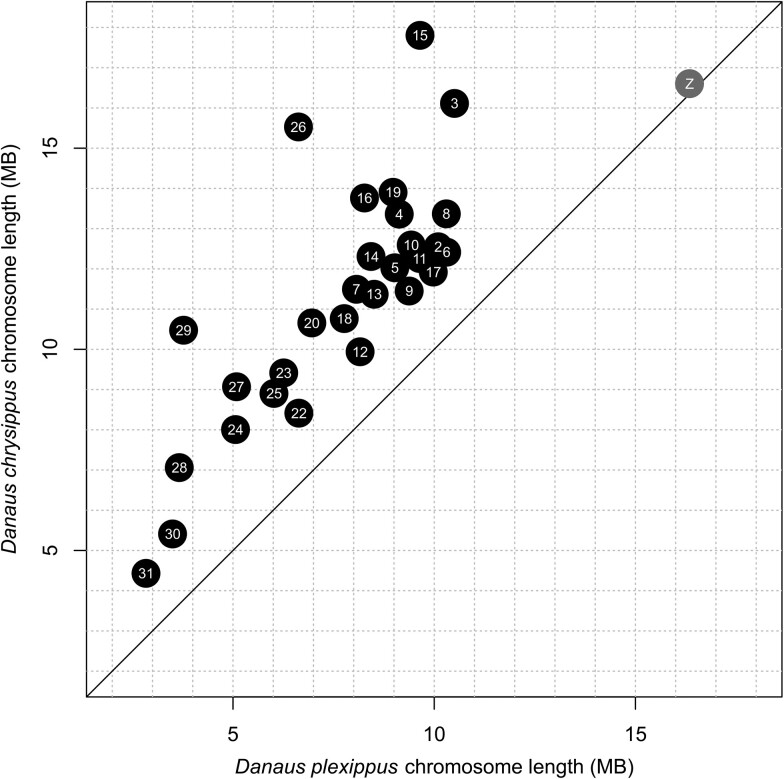
Chromosome length comparison between *D. chrysippus* and *D. plexippus* (MEX_DaPlex assembly). Chromosome lengths represent the sum total of the contigs/scaffolds assigned to each chromosome. Autosomes are shown in black and the Z sex chromosome in grey.

In total, the 354 Mb *D. chrysippus* genome is 42–44% larger than that of *D. plexippus* (245–248 Mb). This difference is consistent for all autosomes, but most dramatic for the 3 chromosomes carrying large repetitive/duplicated tracts: namely 15, 26, and 29 (Fig. 2). By contrast, the Z sex chromosome is nearly identical in size in the 2 species. This difference in autosome sizes could be explained either via a systematic size reduction in the lineage leading to *D. plexippus*, or a systematic increase in the lineage leading to *D. chrysippus*. These hypotheses can be distinguished by comparison with assemblies of other members of the genus or outgroups in the future.

### Transposable element and repeat content

**Fig. 3.**
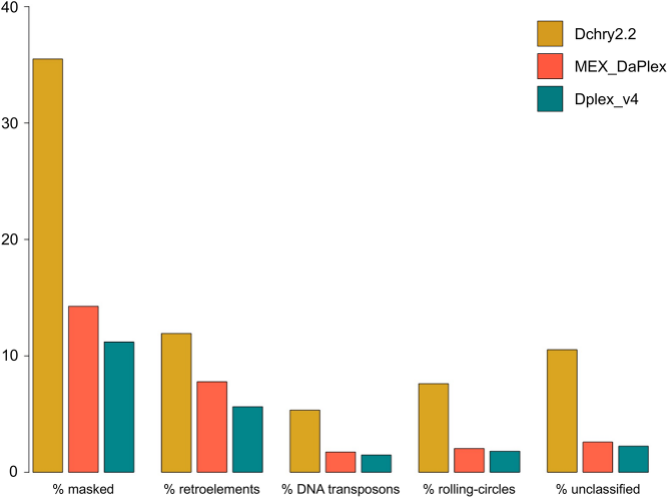
Barplot showing the proportion of the genome of *Danaus chrysippus* (yellow) and *D. plexippus* (represented by both the MEX_DaPlex, in red, and Dplex_v4, in turquoise, assemblies) made up of repetitive elements (as identified and masked by repeatmodeler and repeatmasker). Additionally, the proportion of each genome made up of specific repetitive element families is shown highlighting the increased proportion of repetitive elements in *Danaus chrysippus*.

**Fig. 4.**
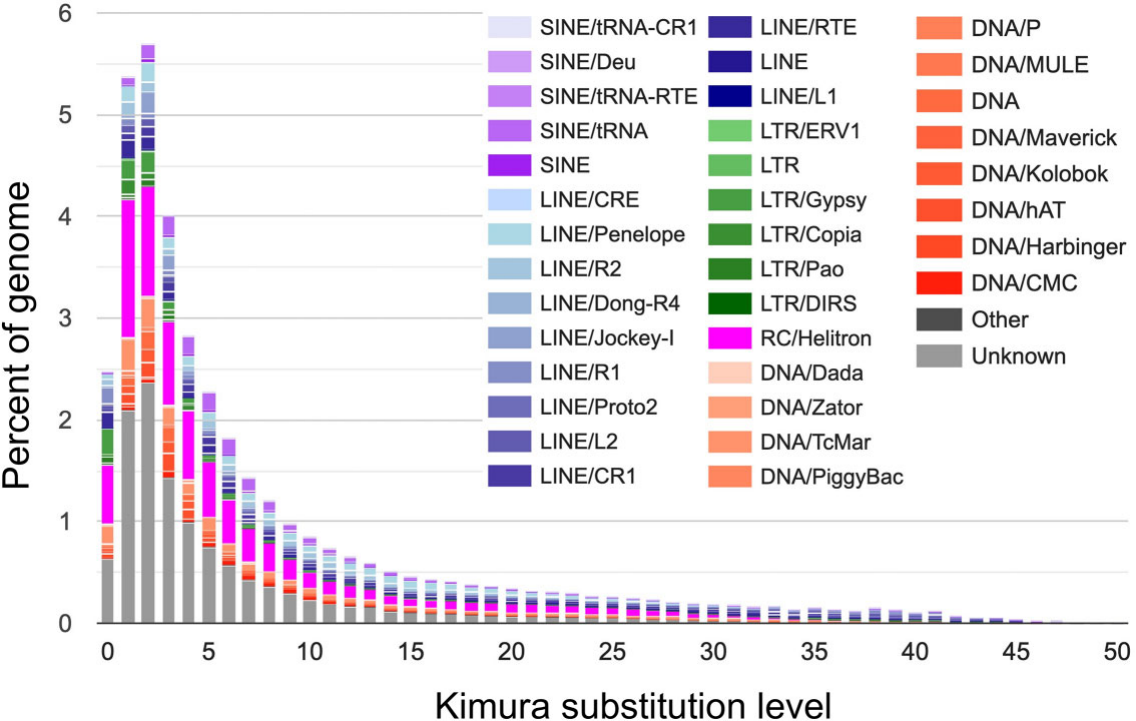
The repeat landscape of the Dchry2.2 assembly. In addition to unclassified repeats, rolling-circle (RC/Helitron), LINE and LTR families all appear to have expanded recently.

In total, the *D. chrysippus* genome comprises 35.5% repeats, with the largest proportion of these being retroelements which make up 11.9% of the genome sequence (Fig. 3). Repeat masking of each of the *D. plexippus* assemblies revealed that a substantially lower proportion of the genomes of these close relatives comprise repeats, only between 11.2% and 14.3%. Each of the main classified repeat families is more abundant in *D. chrysippus* compared to *D. plexippus*, with the largest difference between the species observed for the rolling-circle family which represents 1.8% and 2% of the *D. plexippus* genome sequence, compared to 7.6% of the *D. chrysippus* sequence. The repeat landscape of *D. chrysippus* (Fig. 4) highlights a number of expanding repeat families, most strikingly the rolling-circle repeats RC/Helitron (pink; Fig. 4). The increased prevalence of repetitive elements within the *D. chrysippus* genome (91–98 Mb more than *D. plexippus*) largely explains the larger genome size of *D. chrysippus* compared to *D. plexippus* (an increase of 106–109 Mb). Although the repeat content of genomes across the Lepidoptera order has been shown to vary substantially (Talla et al. 2017), our results suggest that even within a genus a large amount of variation can be present. Although the genome of *D. chrysippus* is rather repetitive, even within Lepidoptera (With repeats making up 35.5% of the genome), *D. plexippus* tends toward the lower end of repeat content (repeats make up 11.2–14.3% of the genome).

### Gene content

In total 16,260 protein-coding genes were annotated in the assembly by BRAKER2 (with 19,639 protein-coding mRNAs annotated—accounting for multiple transcripts/isoforms of the same gene), which included 136,694 exons and 117,106 introns. This number of genes is similar to that of the published annotations for each of the *D. plexippus* assemblies, which annotated 15,006 (Dplex_v4) and 15,995 (MEX_DaPlex) genes (as well as our re-annotated versions of these assemblies which annotated 18,663 and 21,311 genes; in both cases, our annotation involved annotating additional smaller scaffolds not annotated in the original assemblies—284 vs. 30 scaffolds for Dplex_v4 and 64 vs. 55 for MEX_DaPlex). An analysis of BUSCOs using the *insecta_odb10* benchmarking set shows that the full-genome sequences and annotated protein set for *D. chrysippus* are 98.2% and 96.3% complete for BUSCOs, respectively. This percentage completeness is close to that of both published *D. plexippus* annotations which have 94.6% (Dplex_v4) and 97.1% (MEX_DaPlex) complete BUSCOs (Table 1). Pannzer2 allowed us to add functional annotation to 9,567 of the full 16,260 gene set (functional annotation available at https://doi.org/10.5281/zenodo.5731560).

**Table 1.** BUSCO scores for the *D. chrysippus* genome assembly and each *D. plexippus* assembly in addition to the protein sequences resulting from both the original and re-annotation of each of these assemblies (using the insecta_odb10 BUSCO set, *n* = 1,367).

		Complete (%)	Single (%)	Duplicated (%)	Fragmented (%)	Missing (%)
Genomes	Dchry2.2	98.2	97.5	0.7	0.6	1.2
	Dplex_v4	98.7	98.2	0.5	0.7	0.6
	MEX_DaPlex	98.9	98.2	0.7	0.4	0.7
Protein sets	Dchry2.2	96.3	79.7	16.6	1.1	2.6
	Dplex_v4	94.6	93.7	0.9	2.5	2.9
	Dplex_v4 (re-annotated)	98.1	72.6	25.5	1.2	0.7
	MEX_DaPlex	97.1	87.1	10.0	1.3	1.6
	MEX_DaPlex (re-annotated)	98.8	72.6	26.2	0.7	0.5

### Intron and exon length

**Fig. 5.**
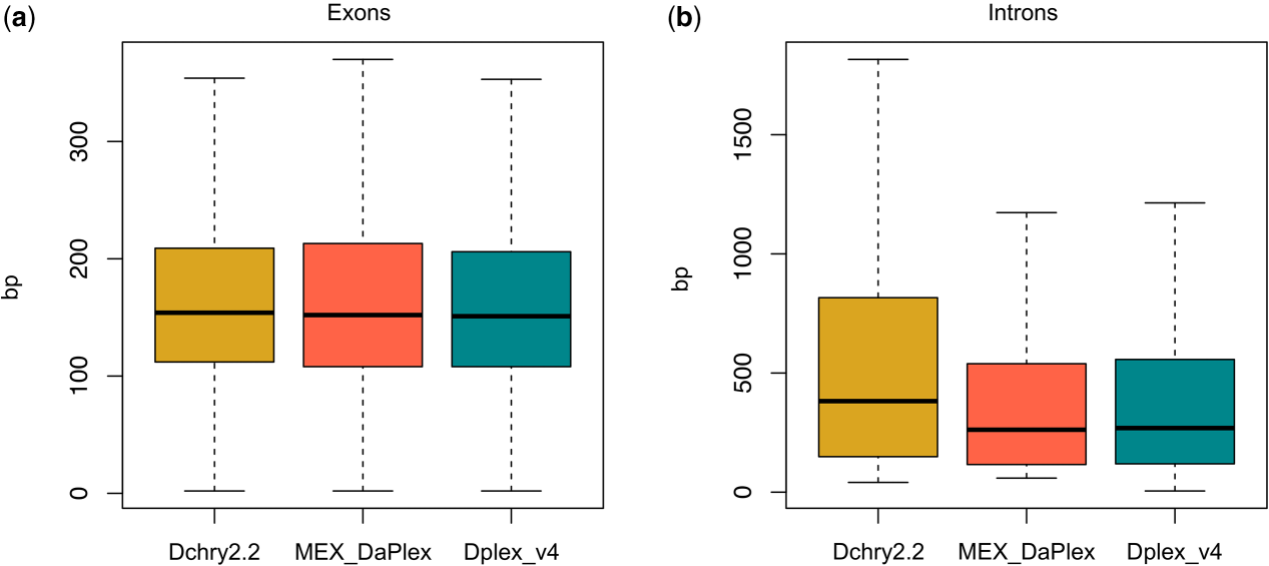
Boxplots showing the distribution of (a) exon lengths and (b) intron lengths taken from the longest transcripts annotated with BRAKER2 for the 3 assemblies, *D. chrysippus* (Dchry2.2) and each of the re-annotated *D. plexippus* assemblies (MEX_DaPlex and Dplex_v4). Outlier points were omitted for clarity. Mean exon length is 226 bp in Dchry2.2, 238 bp for the re-annotated MEX_DaPlex assembly, and 217 bp for the re-annotated Dplex_v4 assembly. Mean intron length is 975 bp in Dchry2.2, 665 bp for the re-annotated MEX_DaPlex assembly, and 738 bp for the re-annotated Dplex_v4 assembly.

Exon length is relatively consistent across the 3 genomes, ranging from 217 bp (Dplex_v4 re-annotation) to 238 bp (MEX_DaPlex re-annotation; Fig. 5a). However, the mean intron length in the *D. Chrysippus* assembly (975 bp) is higher than that in the 2 *D. plexippus* assemblies (665 and 738 bp, respectively; Fig. 5b). This substantial increase in intron length in *D. chrysippus* likely explains the remaining variation in genome size between *D. chrysippus* and *D. plexippus*. This difference may represent a neutral increase in introns in *D. chrysippus* or a selection-mediated reduction in intron size in *D. plexippus*. These hypotheses may be resolved by comparison with genomes of other members of the genus in the future.

## Conclusions

We have assembled a nearly chromosome-level genome for *D. chrysippus*, which is highly comparable in its quality to the best available assembly for *D. plexippus*. Although the 2 species retain strong synteny, the *D. chrysippus* genome is >40% larger, with more repetitive content and larger introns on average. This implies stronger selection to limit nonessential DNA in *D. plexippus*. Future comparative studies involving other members of the genus could shed light on the processes and forces driving the evolution of genome size. The *D. chrysippus* genome will also serve as a reference for population genomic studies to test hypotheses about the evolution of warning coloration, host–parasite interactions, and the consequences of chromosomal rearrangements.

## Supplementary Material

jkab449_Supplementary_Data

## Data Availability

The assembly and annotation are available at the European Nucleotide Archive project accession: PRJEB47812. Additional data files are provided at https://doi.org/10.5281/zenodo.5731560: purged haplotigs, assembly before manual edits, details of manual edits made to the assembly, and repeat library and functional annotation files. Scripts for genome assembly are available at https://github.com/kumarsaurabh20/DChry2.1 (last accessed 5 October 2021) and scripts for the genome annotation and analysis of introns and exons at https://github.com/RishiDeKayne/Danaus_Dchry2.2_annotation (last accessed 5 October 2021). Supplemental material is available at *G3* online.
