# Supplementary material for: Genome assembly of Danaus chrysippus and comparison with the Monarch Danaus plexippus
Source: G3 (Bethesda). 2021 Dec 31;12(3):jkab449. doi: 10.1093/g3journal/jkab449 (PMC9210279; doi:10.1093/g3journal/jkab449)
Supplement: jkab449_Supplementary_Data [file jkab449_supplementary_data.pdf]

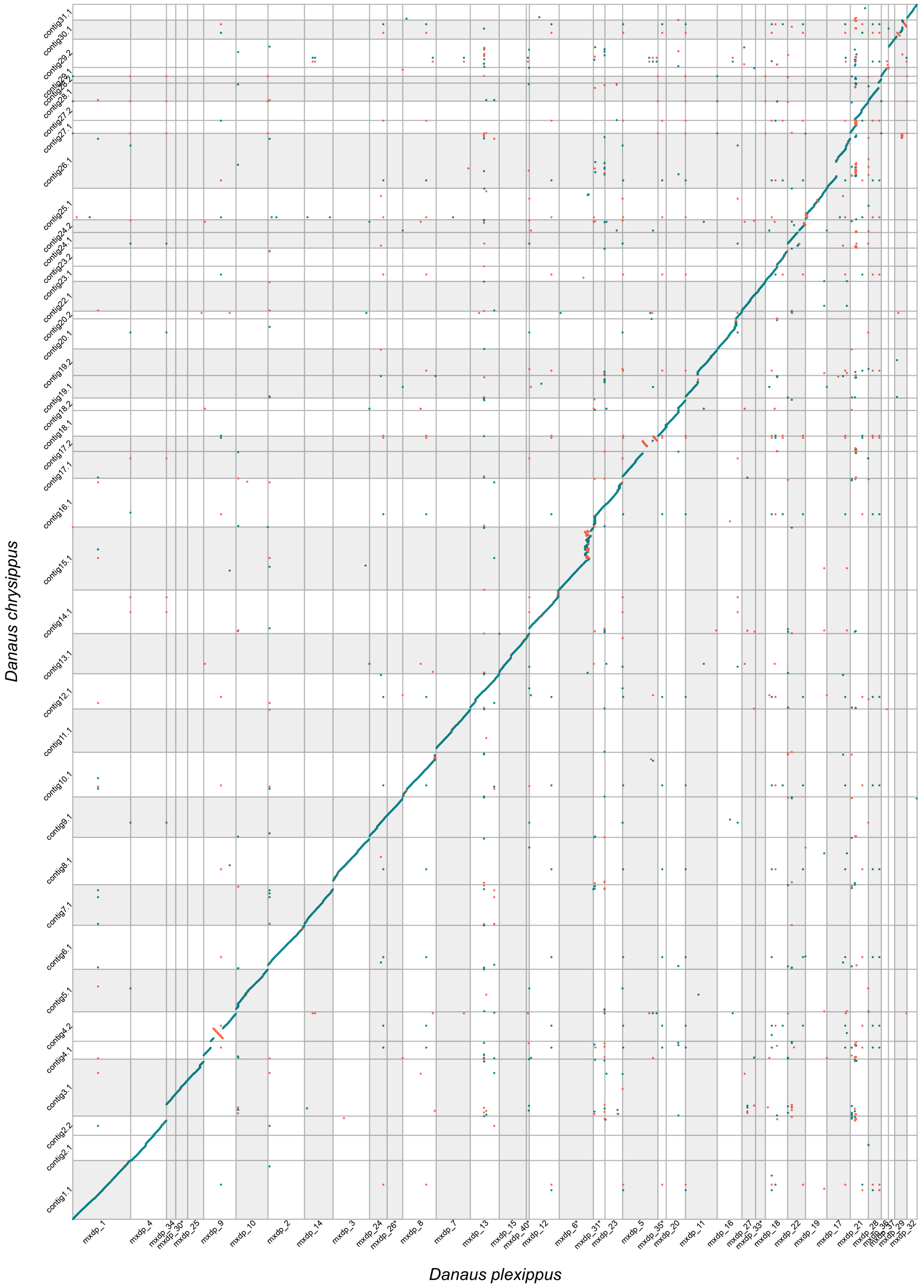

Figure S1. Whole genome alignment between *D. chrysippus* and *D. plexippus* (MEX_DaPlex assembly) showing contig and scaffold labels. Points represent minimap2 alignments greater than 5kb in length. Alignments in the same orientation are shown in turquoise and those in the reverse orientation are shown in red. Only contigs/scaffolds that were confidently assigned to chromosomes (97% of the total in both assemblies) are included. Chromosomes are shaded alternately in grey and white. *D. plexippus* scaffolds that were reversed for ease of visualisation are indicated by an asterisk after the label.
